# Reply to the Letter to the Editor by D. D’Arcangelo et al.: “Ion Channels in Brain Metastasis”—Ion Channels in Cancer Set up and Metastatic Progression Ion Channels in Brain Metastasis

**DOI:** 10.3390/ijms18040719

**Published:** 2017-03-28

**Authors:** Lukas Klumpp, Efe C. Sezgin, Franziska Eckert, Stephan M. Huber

**Affiliations:** 1 Department of Radiation Oncology, University of Tübingen, 72076 Tübingen, Germany; lukas.klumpp@med.uni-tuebingen.de (L.K.); efe.sezgin@uni-tuebingen.de (E.C.S.); franziska.eckert@med.uni-tuebingen.de (F.E.); 2 Dr. Margarete Fischer-Bosch-Institute of Clinical Pharmacology, 70376 Stuttgart, Germany

## Reply:

In their comment on our review article entitled Ion Channels in Brain Metastasis, Dr. D’Arcangelo et al. describe their recent report on aberrant ion channel expression in several cancer entities in order to provide information complementary to that which is presented in our article [1]. In particular, they mention nine transporters/regulators that were found to be up-regulated in tumor specimens in at least half of the analyzed data sets, suggesting a specific oncogenic function of these transporters/regulators [2]. Finally, Dr. D’Arcangelo et al. stress the emerging attention that ion channels are attracting in the oncology field. As a matter of fact, increasing and overwhelming pieces of evidence indicate the functional significance of ion channels in neoplastic transformation of normal cells into cancer cells, malignant progression, metastasis, and therapy resistance of tumor cells [3,4,5]. Our knowledge about the molecular mechanisms of oncogenic ion channels functions, however, lags far behind that of oncogenic signaling by e.g., membrane receptors or kinases. One possible reason is the highly time consuming and elaborate methodology such as patch-clamp recording and Fura-2 Ca^2+^ imaging. These techniques have to be applied in living cells to analyze ion channel function, their regulation, and their downstream signaling. In addition, the comprehensive integration of ion channel activity in tumor biology requires a highly interdisciplinary approach combining (electro-)physiological with cell biological, biochemical and molecular biological expertise. Moreover, for data interpretation, a detailed knowledge about tumor biology and biological effects of anti-tumor therapies such as chemotherapy and radiotherapy is indispensable. As pointed out in the comment of Dr. D’Arcangelo et al., “channelomics” of human tumors, in particular combined with clinical data, certainly complement in vitro and preclinical in vivo studies. While the latter two provide the proof-of-concept for oncogenic ion channel function, omics data may identify proposed oncochannels as independent prognostic or predictive tumor markers in multivariate analysis of patient outcome data. Furthermore, omics, clinical and preclinical data in concert may ultimately lead to the development of ion channel-targeted therapies as promising new strategies of anti-cancer treatment. Importantly, a significant percentage of FDA-approved drugs are ion channel modulators, suggesting the feasibility of those ion channel-targeted therapies. In summary, as emphasized by the comment of Dr. D’Arcangelo et al., ion channel function has a high impact on tumor biology and is thus worth addressing further in the future.
